# The impact of histological type on the accuracy of preoperative N staging in patients with gastric cancer

**DOI:** 10.1186/s12957-019-1674-9

**Published:** 2019-08-01

**Authors:** Atsushi Yamamoto, Yoshihiko Kawaguchi, Kensuke Shiraishi, Hidenori Akaike, Hiroki Shimizu, Shinji Furuya, Naohiro Hosomura, Hidetake Amemiya, Hiromichi Kawaida, Makoto Sudo, Hiroshi Kono, Daisuke Ichikawa

**Affiliations:** First Department of Surgery, Faculty of Medicine, University of Yamanashi, 1110 Shimokato, Chuo, Yamanashi 4093898 Japan

**Keywords:** Histological type, Gastric cancer, Lymph node metastases

## Abstract

**Background:**

The low accuracy of preoperative diagnosis of lymph node metastasis in gastric cancer (GC) complicates decisions on patient indication for neoadjuvant chemotherapy.

**Methods:**

We investigated the use of preoperative clinical diagnosis of lymph node involvement (cN) in GC patients compared with postoperative pathological diagnosis.

**Results:**

In a series of 265 patients enrolled at the University of Yamanashi Hospital, the overall sensitivity was 44.4% and specificity was 93.4% of CT for detecting lymph node metastasis. The positive and negative predictive values were 80.0% and 73.8%, respectively. The negative predictive value was lower for undifferentiated adenocarcinoma than that for differentiated adenocarcinoma (64.9% vs. 78.7%, *p* = 0.034). In cT2 ≤ and cN2 ≤ GC, overdiagnosis of lymph node metastasis was significantly more frequent in patients with differentiated (50.0%) than in undifferentiated (13.3%) adenocarcinoma (*p* = 0.046).

**Conclusions:**

Diagnostic accuracy of lymph node involvement depended on histological type and cT-stage. Thus, considering preoperative histological type in GC, it may be useful to decide treatment plan.

## Introduction

Gastric cancer (GC) is a leading cause of cancer-related deaths worldwide. Although the frequency of early GC diagnosis has increased due to the recent advancements in diagnostic tools, the therapeutic outcomes of advanced GC remain unsatisfactory. Radical gastrectomy followed by adjuvant chemotherapy is a standard treatment for patients with locally advanced GC and has improved the outcomes in some patients, but the prognosis of advanced GC with extensive lymph node metastases remains dismal. Among the classifications used worldwide, the nodal status is recognized as one of the most important prognostic factors. Tsuburaya et al. recently reported that neoadjuvant chemotherapy (NAC) followed by radical gastrectomy improved the survival rate of patients with advanced GC with bulky metastatic lymph nodes along the major perigastric vessels and/or the aorta in a clinical trial [1]. However, GC may progress in some patients during the delivery of NAC. Therefore, it is important to avoid false indication of NAC and identify patients who are not likely to respond to NAC, but who would have a relatively favorable prognosis following standard treatments, operation, and postoperative chemotherapy. Various imaging methods have been attempted for preoperative diagnosis of metastatic lymph nodes, of which multidetector-row computed tomography (MDCT) would be the most useful strategy for accurate preoperative diagnosis of lymph node stage in clinical settings. This retrospective study investigated the usefulness of MDCT to predict lymph node staging in GC patients by comparing the clinical and pathological status (cN and pN) and assessed the results of comparison based on clinical tumor depth and the histological type.

## Materials and methods

### Patients

Between January 2013 and May 2018, 283 patients with GC underwent surgical resection at the University of Yamanashi Hospital. Patients who did not undergo gastrectomy (who underwent bypass or pancreatoduodenectomy among others) and those without radical lymph node dissection were excluded. All patients were evaluated preoperatively by abdominal MDCT (Aquilion ONE™, Canon), 130 patients with an image thickness of 5 mm, the 149 patients with an image thickness of 1 mm, and remaining patients with an image thickness of 3, 2, or 0.5 mm. There were 268 patients with contrast-enhanced CT images and 15 patients with simple CT images. A total of 265 patients were included in this retrospective study. Patients underwent gastrectomy with D1, D1+, or D2 lymph node dissection according to the fourth version of the Japanese Gastric Cancer Treatment Guidelines [2]. There was a term form 1 day to 5 months between CT examination and surgery. The extent of gastric resection was generally determined by the tumor location. The clinicopathological features of each patient were retrieved from the hospital database. Tumor specimens and dissected lymph nodes were obtained during surgery and embedded in paraffin. The macroscopic and microscopic classification of the tumors was based on the Japanese Classification of Gastric Cancer (JCGC; 15th edition). This study was approved by the Ethics Committee of the University of Yamanashi Hospital and was performed in accordance with the ethical standards of the Declaration of Helsinki [3] and its later amendments.

### Evaluation of lymph node status

Contrast-enhanced CT images were obtained during the portal venous phase after intravenous administration of nonionic contrast material. Lymph nodes with either a minor axis of ≥ 8 mm or a major axis of ≥ 10 mm were diagnosed as positive, i.e., metastatic, by their appearance on CT images. The number and location (station numbers) of enlarged lymph nodes were recorded. The clinical lymph node stage (cN) was recorded preoperatively and compared with the subsequent postoperative pathological stage (pN) diagnosis. Each stage of lymph node metastasis was classified based on JCGC determined by the number of metastatic lymph nodes as follows: stage N1 included 1–2 affected nodes, N2 included 3–6 nodes, and N3 included > 6 affected nodes. Further comparative analyses were performed for each histological type, differentiated or undifferentiated. Papillary adenocarcinoma and moderately and well-differentiated adenocarcinomas were classified as differentiated. Poorly differentiated adenocarcinoma, signet-ring cell carcinoma, and mucinous adenocarcinoma were classified as undifferentiated.

### Statistical analysis

Statistical analysis was performed with EZR (Saitama Medical Center, Jichi Medical University, Saitama, Japan), which is a graphical user interface for R (The R Foundation for Statistical Computing, Vienna, Austria; https://www.r-project.org/foundation/). Quantitative results were presented as means ± SD and evaluated with Student’s *t* test or the Mann–Whitney *U* test. Qualitative results were evaluated with the *χ*^2^ test or Fisher’s exact test as appropriate. *p* values < 0.05 were considered statistically significant.

## Results

### Comparison of cN and pN status

The clinicopathological characteristics of patients are summarized in Table 1. Forty-four (80.0%) of the 55 patients with a clinical diagnosis of lymph node metastasis on preoperative CT had pathologically confirmed lymph node metastasis. Fifty-five of 210 patients (26%) diagnosed preoperatively as cN0 were found to have pathologically confirmed metastases. The sensitivity of contrast-enhanced MDCT for lymph node metastasis was 44.4% (44/99), and the specificity was 93.4% (155/166). Further detailed analyses were conducted for each nodal stage based on the total number of metastatic lymph nodes (N0, N1, N2, and N3) (Table 2). As cN3 was found to be difficult to distinguish from cN2 in our previous study [4], based on the nodes in clinical and pathological stages N2 and N3, patients were grouped into categories cN2 ≤ and pN2 ≤ for further analyses. The detailed clinical nodal analysis provided an accurate diagnosis of the nodal stage in 180 of 265 patients (67.9%). Preoperative overdiagnosis was made in 17 patients (6.4%), and underdiagnosis was made in 68 (25.7%).

**Table 1:** Clinicopathological characteristics of the patients analyzed (*N* = 265)

Characteristic	
Age (years)	69.4 (29–90)
Male	183
Female	82
Papillary adenocarcinoma	4
Well-differentiated adenocarcinoma	96
Moderately differentiated adenocarcinoma	63
Poorly differentiated adenocarcinoma	78
Signet-ring cell carcinoma	22
Mucinous carcinoma	2

**Table 2:** Comparison of clinical and pathological lymph node metastasis

	pN0	pN1	pN2 ≦
All			
cN0	155	27	28
cN1	7	7	13
cN2 ≦	4	6	18
Differentiated			
cN0	107	17	12
cN1	6	4	4
cN2 ≦	3	5	5
Undifferentiated			
cN0	48	10	16
cN1	1	3	9
cN2 ≦	1	1	13

### Comparative results in early (cT1) and advanced (cT2 ≤) gastric cancer

Most early-stage (cT1) patients were diagnosed preoperatively as lymph node metastasis negative (Table 3). Eighteen (12.2%) of 148 cN0 patients were found to be pN positive. Six of nine patients with early cN-positive GC were found to be pN negative (66.7%). Approximately 50% of advanced stage (cT2 ≤) patients had a preoperative cN-positive diagnosis, and 41 (89.1%) of the 46 cN-positive patients were also pN positive. Thirty-seven of 62 advanced-stage patients without cN-diagnosed metastases were found to be node positive on histological evaluation. Overall, underdiagnosis of lymph node metastasis was more frequent than overdiagnosis in patients with advanced-stage GC (45.4% vs. 8.3%, respectively).

**Table 3:** Comparison of the differentiated and undifferentiated tumors in early stage

	pN0	pN1	pN2 ≦
cT1, all			
cN0	130	12	6
cN1	5	0	1
cN2 ≦	1	2	0
cT1, differentiated			
cN0	93	8	3
cN1	5	0	0
cN2 ≦	1	2	0
cT1, undifferentiated			
cN0	37	4	3
cN1	0	0	1
cN2 ≦	0	0	0

### Comparative results by histological type

Histologically differentiated adenocarcinomas were diagnosed in 163 patients; 102 patients had undifferentiated adenocarcinomas (Table 1). The difference in tumor histology was significant in patients with advanced GC, with 55.9% of patients diagnosed with undifferentiated and 31.2% diagnosed with differentiated adenocarcinoma (*p* < 0.001). The positive predictive value for detecting lymph node metastasis was higher for undifferentiated (92.9%) than differentiated adenocarcinoma (66.7%) (*p* = 0.059). The negative predictive value was significantly higher for differentiated (78.7%) than for undifferentiated (64.9%) adenocarcinoma (*p* = 0.036). Analysis by nodal stage found that accurate nodal diagnoses were made in 116 of 163 patients with differentiated adenocarcinoma (71.2%) and in 38 of 102 patients with undifferentiated adenocarcinoma (37.3%) (*p* = 0.020). The difference in the accuracy of diagnosis might have resulted from the large number of early GCs that were differentiated adenocarcinomas. However, in the entire cohort, overdiagnosis was more frequent in patients with differentiated adenocarcinoma (8.6%) than in those with undifferentiated adenocarcinoma (2.9%) (*p* = 0.076). In patients with advanced GC (T2 ≤ tumor), overdiagnosis was also significantly more frequent in patients with differentiated adenocarcinoma and cN2 ≤ lymph node metastases (50.0%) than in those with undifferentiated adenocarcinoma (13.3%) (*p* = 0.046) (Table 4).

**Table 4:** Comparison of differentiated and undifferentiated tumors in advanced stage

	pN0	pN1	pN2 ≦
cT2 ≦, all			
cN0	25	15	22
cN1	2	7	12
cN2 ≦	3	4	18
cT2 ≦, differentiated			
cN0	14	9	9
cN1	1	4	4
cN2 ≦	2	3	5
cT2 ≦, undifferentiated			
cN0	11	6	13
cN1	1	3	8
cN2 ≦	1	1	13

## Discussion

Lymph node metastasis has a significant influence on the prognosis of patients with GC, and various methods are used to identify and determine the number of metastatic lymph nodes, including ultrasonography, endoscopic ultrasound sonography, CT, magnetic resonance imaging, and positron emission tomography (PET). Among these, MDCT is accepted as the most powerful diagnostic method. Preoperative diagnosis is made mainly by node size rather than shape or the enhanced contrast pattern. Node size is easy to obtain and is a reproducible criterion. It can also be obtained by simple CT without contrast enhancement in patients with renal dysfunction or atopy. The cutoff values used in this study, a minor axis of ≥ 8 mm or a major axis of ≥ 10 mm, are commonly used for the diagnosis of metastasis. The sensitivity was 44.4%, and the positive predictive value was 80.0%; these are consistent with the results of previous reports [5–7]. Although sensitivity may differ depending on the location of the tumor and the nodes, we did not consider it in this study. Fukagawa et al. investigated the diagnostic accuracy of lymph node metastasis by CT in a clinical trial, with the same size criteria as that used in this study, and found that the sensitivity and positive predictive values were 62.5% and 77.7%, respectively [8]. In clinical practice, PET can be used to diagnose lymph node metastasis, especially metastasis of distant nodes. However, lymph nodes near the primary tumor are sometimes difficult to diagnose accurately, and the histological type can influence their detection. The sensitivity of PET scans may be decreased in histologically diffused GC tumors because of low glucose transporter 1 (GLUT1) expression that reduces fluorodeoxyglucose uptake. Therefore, the usefulness of the routine preoperative PET evaluation for the diagnosis of lymph node metastasis is limited [9]. Also, PET may help us diagnose the lymph node metastases accurately, but in this study, not all of the patients have diagnosed with these modalities; thus, we have considered only with CT.

There were quite few reports about the relationship between histological types and lymph node involvement in GC. In this study, we considered that preoperative histological types would help us make a decision on how to treat patients with severe GC. Recently, preoperative chemotherapy has been attempted to further improve the prognosis of patients with advanced GC. Several clinical trials have been conducted to evaluate the efficacy of NAC in patients with GC, and in Japanese gastric cancer treatment guidelines 2018 (ver. 5), NAC is recommended for patients with extensive, bulky lymph node metastasis [1, 10–12]. However, cT1/T2 tumors may be mostly cured safely by initial surgery without preoperative chemotherapy, and prolonged NAC is occasionally accompanied by disease progression [8]. Teranaka et al. recently reported that tub2 and pap show higher incidence of lymph node metastasis than tub1, so individualization of tub1, tub2, and pap is important when they consider endoscopic treatment [13]. There was no literature that has been shown about the relationship between histological types and lymph node involvement, but in this study, the size of metastatic lymph node of differentiated adenocarcinoma might tend to be larger than that of undifferentiated adenocarcinoma because there were a lot of overdiagnosis cases. This speculation needs to be clarified in further studies. We evaluated diagnostic accuracy by histological type and found that only patients with advanced undifferentiated GC and cN2 ≤ or bulky metastases are likely to benefit from aggressive treatment with NAC. Precaution would avoid a false indication of the potential benefit of NAC in patients who would have a favorable prognosis following standard treatment, surgery, and postoperative chemotherapy. Thus, we considered that if the accuracy of the diagnosis of lymph node metastases would be improved, NAC may be effective. Further studies with large patient cohorts should be conducted to confirm these results.

## Conclusion

Preoperative CT diagnosis of lymph node involvement provided useful clinical information on lymph node metastasis. Diagnostic accuracy depended on the histological type and cT-stage.

Thus, considering preoperative histological type in GC, it may be useful to decide the treatment plan.

## Data Availability

All data generated or analyzed during this article are included in this published article.
